# A Systematic Review of Whether the Use of N95 Respirator Masks Decreases the Incidence of Cardiovascular Disease in the General Population

**DOI:** 10.7759/cureus.29823

**Published:** 2022-10-02

**Authors:** Ameer Khan, Munir A Khan, Matthew Perry, Udai K Godhania, Omer J Khan, Aftab A Khan

**Affiliations:** 1 Cardiology, Tameside General Hospital, Ashton-Under-Lyne, GBR; 2 Cardiology, University of Leeds, Faculty of Medicine, Leeds, GBR; 3 Psychiatry, Faculty of Medicine, University of Craiova, Craiova, ROU; 4 Surgery, Fauji Foundation Hospital, Lahore, PAK

**Keywords:** public health education, cardiovascular effects, cardiovascular implications, cardiovascular prevention, n95 respirators

## Abstract

The usage of masks such as the N95 has increased exponentially worldwide. With the ever-increasing global rates of cardiovascular disease, it is vital that preventative measures are adopted to help tackle this crisis. N95 masks have been promoted as health prevention odysseys in the battle against viruses such as COVID-19. A systematic review was conducted on whether the N95 masks could help improve our cardiovascular health. Our data sources included PubMed, Medline and Scopus. Eleven studies met the eligibility criteria to be included in the review. N95 mask usage led to increased reports of dyspnoea, however, no significant effect was seen on blood pressure. N95 masks also showed improvement in aortic parameters. While encouraging results were yielded, further focussed studies on the use of N95 masks and the effect on various cardiovascular parameters would help strengthen the association.

## Introduction and background

The seismic impact of the coronavirus disease 2019 (COVID-19) pandemic has had an enormous effect on all walks of life. One of the biggest interventions of the pandemic has been the rising use of various face coverings to minimise viral transmission. The wide extent of usage during the height of the pandemic can be demonstrated by the fact that up to 90% of people worldwide lived in areas where the use of face masks was deemed mandatory or they were universally used in certain public areas [1].

Research has proven the efficacy of face masks, with studies showing an estimated 70% reduction in COVID-19 infection in healthcare workers with mask use [2]. Various types of masks have been used across the globe, with the N95 being one of the most common [3]. The N95 respirator has been proven to be the most effective face covering in reducing the transmission of COVID-19 [3]. The mask works by providing a protective barrier, reducing the ability of pathogenic airborne particles from penetrating our body's defences [3].

The N95 masks have proven to be effective in controlling the spread of other diseases, including measles, tuberculosis and chicken pox, demonstrating the versatility and profound impact they have in reducing respiratory disease transmission [4].

With global industrialisation, the demand for energy has increased to an all-time high and thus has led to a significantly increased release of emissions. There has been compelling evidence to suggest that these air pollutants can have a detrimental effect on health, especially cardiovascular-related outcomes, including mortality [5].

With rising air pollution combined with the impact these pollutants have on cardiovascular health, significant preventative measures are needed to combat this threat and help reduce the burden of cardiovascular disease on our global population [6].

The need for preventative cardiovascular medicine is at an all-time high, providing valuable insight into easily achievable lifestyle modifications that could reduce morbidity, mortality and healthcare costs. Since N95 respirators have shown to be amongst the most effective face coverings, this systematic review aims to answer the question of whether the N95 respirator can improve cardiovascular health.

## Review

Methods

In order to conduct this review, a robust search was performed to find the most relevant papers pertaining to the subject. An electronic literature search was conducted using the databases MEDLINE, PubMed and SCOPUS on the 12th of July 2022. The search terms used were “N95 mask or face mask or N95 or medical mask or surgical mask” AND “cardiovascular disease or CVD or heart or cardiac or coronary heart disease”. Outcomes related to cardiovascular health included, but were not limited to, heart rate, blood pressure, dyspnoea, end-tidal carbon dioxide, oxygen saturation and exercise tolerance. Boolean operators were utilised to help conduct a thorough search of the literature available. The search terms were used as keywords and as MeSh terms to facilitate a broad-based search. Titles and abstracts were screened for inclusion. Papers were excluded if they were not written in English, if they were review papers, if they did not contain data on the use of N95 masks, and if they did not contain data relevant to cardiovascular disease. A restriction was posed on the date of publication as being within the last 10 years due to the increasing prominence of usage of face masks during this period of time heightened by the COVID-19 pandemic.

**Figure 1:**
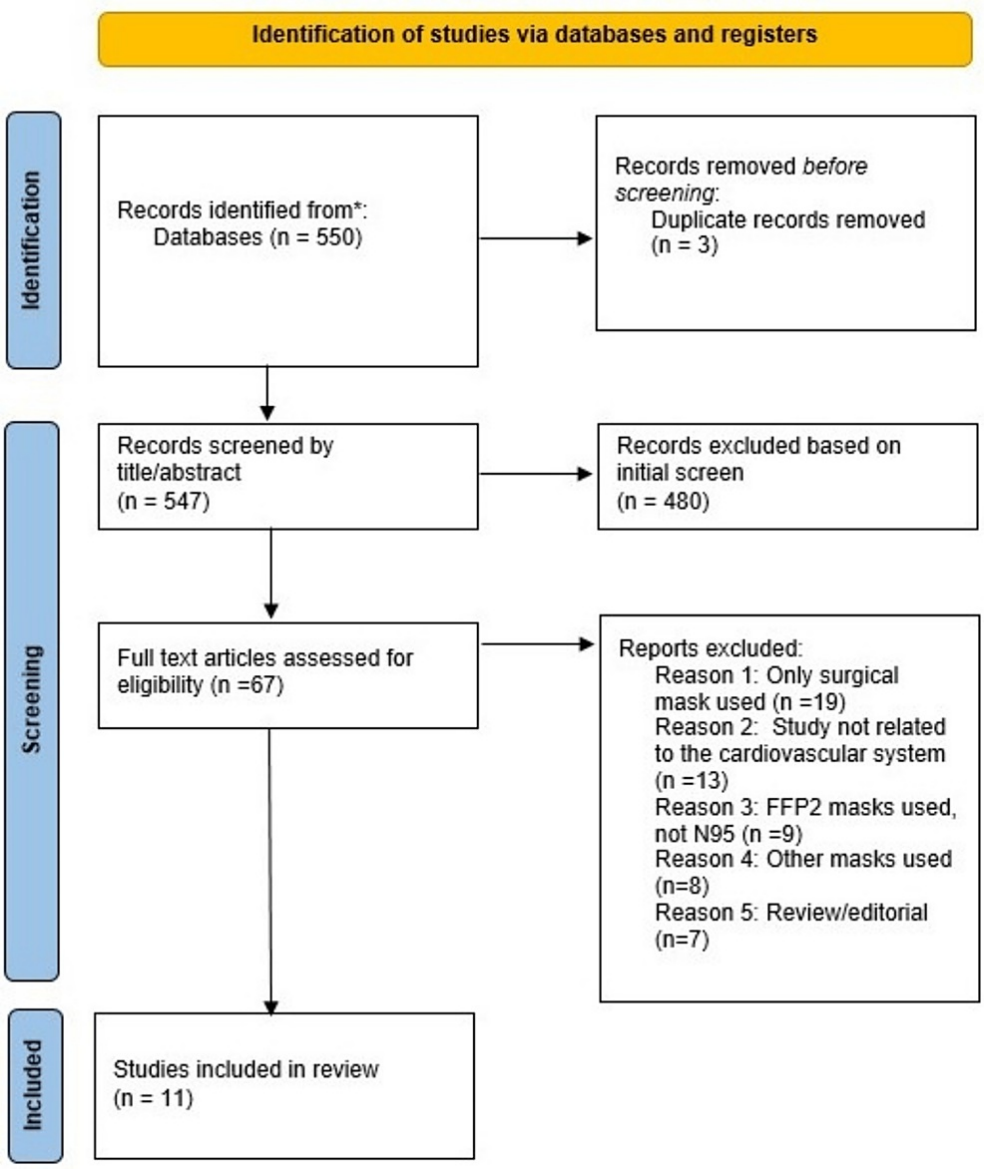
PRISMA Flowchart PRISMA: Preferred Reporting Items for Systematic Review and Meta-Analysis

All relevant articles were scanned by three authors. All papers chosen to be included in the review underwent a final consensus between the three authors followed by a final re-evaluation of the chosen literature. This systematic review was completed per Preferred Reporting Items for Systematic Review and Meta-Analysis (PRISMA) [7]. The PRISMA flowchart in Figure 1 summarises our search below.

Results

Table 1 summarises the 11 papers chosen for the purpose of this review and the key aspects of data extracted from the paper pertaining to the use of masks and cardiovascular disease. The total number of participants involved across the studies selected was 804, which included 505 males and 299 females.

**Table 1:** Results Table Summarising the Papers Selected Within the Review

Name	Author	Year	Face mask used	Key findings	Study design	Sample size & population	Location
Does Wearing a Facemask Decrease Arterial Blood Oxygenation and Impair Exercise Tolerance?	Ade, C. J., et al. [8]	2021	N95 surgical mask, flannel mask	The results supported the hypothesis and found that none of the masks reduced arterial blood oxygenation saturation during rest, sub-maximal or maximal exercise. None of the masks provided any significant changes in primary cardiovascular responses including mean arterial pressure, stroke volume and cardiac output. There was no effect on maximal exercise capacity whilst using any of the masks However, ratings of dyspnoea were significantly increased by wearing a mask as well as an increase in end-expired peri-oral %CO_2_ and reducing %O_2 _during exercise.	Randomized cross-over study	11 participants were involved in the study with 5 men and 6 women. The participants had an average age of 30 ± 11 yrs. They had an average height of 175 ± 11 cm with an average body mass of 73.0 ± 12.9 kg. All the individuals were experienced with laboratory exercise testing and maximal exercise tests. Participants were known to be free from cardiovascular, pulmonary or metabolic disease and were non-smokers with this information obtained from a questionnaire.	USA
Does Wearing a Mask While Exercising Amid the COVID-19 Pandemic Affect Hemodynamic and Hematologic Function Among Healthy Individuals? Implications of Mask Modality, Sex, and Exercise Intensity	Ahmadian, M., et al. [9]	2021	N95 surgical mask	No significant difference in haemodynamic parameters including heart rate, systolic blood pressure and rate pressure product between the three groups or any significant haematological changes following exercise. Heart rate was significantly elevated in females who used the N95 mask one-minute post-exercise compared to the no mask group.	Randomised cross-over study	144 participants with 72 males and 72 females, all non-smokers. All participants were aged over 20 and had no background of chronic illness including arthritis diabetes, hypertension, cancer, heart attack, chronic cough and bronchitis. They also had a haemoglobin level equal to or greater than 11g/dl. All of those involved in the study had normal levels of arterial oxygenation with a saturation of over 95%.	Iran
Return to Training in the COVID-19 Era: The Physiological Effects of Face Masks During Exercise	Epstein, D., et al. [10]	2021	N95 Surgical mask	Systolic blood pressure at exhaustion did not vary significantly between the study groups (143 ± 14 mm Hg without a mask, 143 ± 16 mm Hg with a surgical mask and 147 ± 16 mm Hg with N95). The differences in heart rate, respiratory rate, oxygen saturation, and the rate of perceived exertion did not reach statistical significance at any stage of the study. The use of an N95 mask was associated with higher end-tidal carbon dioxide values during most of the exercise period in comparison to those without a mask.	Multiple cross-over, self-control trial	16 healthy non-smoking young adult males over 18 who participate in either 75 minutes of vigorous aerobic exercise a week or 150 minutes of moderate-intensity exercise a week. Subjects were excluded if had any medical condition exacerbated by strenuous physical exercise including diabetes, respiratory and cardiovascular disease and any acute respiratory illness two weeks prior to the tests. The mean age of the participants was 34 ± 4 years. The mean height, weight, and BMI were 179 ± 7 cm, 76.3 ± 11.8 kg, and 28.72 ± 3.78 kg/m^2^, respectively.	Israel
Acute Cardiovascular Effects of Traffic-Related Air Pollution (TRAP) Exposure in Healthy Adults: A Randomized, Blinded, Crossover Intervention Study	Han et al. [11]	2021	N95-powered air-purifying respirator	No significant differences from the baseline in heart rate, systolic and diastolic blood pressure following the use of the N95 respirator.	Randomised double-blinded, crossover intervention study	39 healthy university students including 19 males and 20 females. The average age of the participants was 22.7 and the average body mass index was 21.0 kg/m^2^. Study participants were non-smokers and declared that they had no clinically diagnosed cardiovascular disease. All reported staying in the vicinity of the university within 24 hours of the test day.	China
Cardiovascular Evaluation Using Exercise Testing Performed With Face Masks During the COVID-19 Pandemic	Ilarraza-Lomelí et al. [12]	2021	N95	In patients who underwent exercise tests during the pandemic and thus wore an N95 mask, blood pressure levels were higher than in the group of patients who performed their exercise tests before the pandemic. Exercise tests conducted in the pandemic resulted in an increased number of tests suspended due to dyspnoea. Maximum exercise tolerance did not show significant differences between both groups.	Cross-sectional study	A total of 361 stress tests were studied, 209 (58%) belonging to the NO PANDEMIC group and 152 (42%) to PANDEMIC-G. Those in the PANDEMIC-G group used the N-95 masks. This included 132 males and 20 females. The average age of these individuals was 45 ± 21 and the average body mass index was 25.8 ± 5 kg/m^2^.	Mexico
Effects of Wearing an N95 Respirator or Cloth Mask Among Adults at Peak Exercise: A Randomized Crossover Trial	Kampert et al. [13]	2021	N95 cloth mask	Perceived breathing resistance was greater in those who wore a mask, particularly in the N95. The use of both the cloth mask and the N95 produced lower heart rates as well as lower peak exercise oxygen uptake.	Randomised crossover trial	20 participants which included 11 men and 9 women. None of the participants had ever smoked and were all deemed to be healthy and recreationally active. The average age of the women in the study was 35±11 and the average body mass index was 25.1± 4.2. The average age and BMI of the men were 39±11 and 25.0±4.2 respectively.	USA
Comparison of Effects of N95 Respirators and Surgical Masks to Physiological and Psychological Health among Healthcare Workers: A Randomized Controlled Trial	Su, C.-Y., et al. [14]	2021	N95 surgical mask	The N95 respirator group did not increase the risk of physiological burdens, but had higher percentages of incidence for several symptoms, such as shortness of breath, headache, dizziness, talking difficulty, and fatigue in comparison with the surgical mask group.	Randomised controlled trial	68 healthcare workers were involved in the study with 34 using the N95 and the remaining 34 using a surgical mask. In the N95 group, there were 27 males and 7 females. The average age within this group was 41.2 ± 8.5.	Taiwan
Physiological Effects of Wearing N95 Respirator on Medical Staff During Prolong Work Hours in Covid-19 Departments	Shechtman, L., et al. [15]	2022	N95	There was a significant decrease in pH and partial pressure of venous oxygen whilst wearing the N95. No significant change in heart rate, oxygen saturation, bicarbonate levels, partial pressure of venous carbon dioxide and end-tidal carbon dioxide. The overall use of N95 may lead to changes in gas exchange.	Single-centre prospective observational study	41 participants with 20 women and 21 males. The average age was 33.3 ± 5.5 and the median age was 34. 20 participants had pre-existing health conditions which included 12 smokers, 2 individuals with hypertension, 1 with asthma and 1 who was pregnant. Those with uncontrolled health conditions were excluded from the study.	Israel
N95 Respirator Use During Advanced Pregnancy	Roberge, R. J., et al. [16]	2014	N95	Usage of the N95 respirators did not produce a significant response in the variables tested which included heart rate, respiratory rate, oxygen saturation, transcutaneous partial pressure of carbon dioxide, chest wall temperature, aural temperature, and subjective perceptions of exertion and thermal comfort. There was also no significant change in foetal heart rate whilst using an N95.	Case-control study	A sample size of 44 with 22 healthy non-smoking pregnant women who were in the second to mid-third trimester and 22 non-smoking non-pregnant women. The averages for the pregnant group were: gestation 20.6 ± 4.5 weeks, age 28.0 ± 2.9 years, height 166.7 ±5.7 cm, weight 73.8 ±18.5 kg and body mass index,26.8 ±6.0 kg/m^2^. The averages for the non-pregnant demographic control group were age 26.1 ±4.0 years, height 167.5 ±5.9 cm, weight 67.5 ±9.5 kg, and body mass index 24.1 ±3.2 kg/m^2^.	USA
Physiologic and Other Effects and Compliance With Long-Term Respirator Use Among Medical Intensive Care Unit Nurses	Rebmann, T., et al. [17]	2013	N95 Surgical mask	Significant elevation in carbon dioxide levels especially whilst using the N95 mask however, the levels did not reach clinically relevant levels. Subjective symptoms such as headache, perceived exertion, light-headedness and difficulty communicating increased over time of wearing masks. No significant changes in blood pressure, O_2_ levels, perceived comfort, perceived thermal comfort, or complaints of visual difficulties whilst wearing the N95. Compliance remained high whilst wearing the N95 mask during the course of the two shifts analysed.	Longitudinal study	10 non-smoking nurses. 9 female nurses and 1 male nurse. All of the nurses were white and had on average 11 years prior experience of wearing an N95. 90% of participants were deemed at least overweight with a body mass index of over 25 with 50% being classed as obese with a body mass index of over 30. They were free from any symptom or condition which could put them at risk from using the N95 mask including pregnancy, arrhythmias, hypertension, poorly controlled asthma, history of panic attacks or claustrophobia and any seizure history.	USA
Acute Blood Pressure and Cardiovascular Effects of Near-Roadway Exposures With and Without N95 Respirators	Morishita, M., et al. [18]	2019	N95	During the near-roadway period, there were no significant changes in brachial blood pressure or other cardiovascular measures. There were, however, reduced levels of aortic augmentation pressure and aortic pulse pressure during roadside exposure whilst using the N95 mask.	Randomised single-blind crossover study	50 participants who were all non-smokers and lived in non-smoking households and did not have any cardiovascular disease or risk factors including known hypertension, hyperlipidaemia or diabetes mellitus. The study included 36 females and 14 males. The average age of participants was 36 ± 14. The average body mass index was 26.1 ± 4.7. The average blood pressure of participants was 120.0/75.9 ± 13.4/9.4 mm Hg.	USA

Discussion

Our systematic review determined that wearing an N95 mask can reduce the risk of cardiovascular diseases in certain circumstances. Several parameters were explored during the review, which can contribute to the development of cardiovascular disease.

An accelerated heart rate has been identified as a risk factor in the development of cardiovascular disease and has shown significance in all-cause mortality in cardiovascular disease [19]. Studies have shown that, in fact, an increase in heart rate by 10 beats per minute had the potential to increase the risk of cardiac death by up to 20% [19,20].

In this review, one particular study demonstrated that at rest, a mild increase in heart rate by 5.6% was noted whilst wearing an N95 respirator in comparison to not wearing a mask [8]. These findings were replicated in other studies such as by Ilarraza-Lomelí et al. [12]. However, both authors commented that the results were not statistically significant to draw the conclusion that the elevated heart rate was an effect of the N95 mask.

Another study demonstrated that females who had used an N95 respirator following a period of exercise had a greater heart rate one minute post-exercise [9]. Despite the change in heart rate, the results were not deemed significant enough to extrapolate any conclusions about the effects of N95 respirators. Seventy-two females were involved in this particular study and perhaps further research with a larger sample size needs to be conducted to analyse if there is any distinct differences between heart rate in males and females following the use of N95 masks. Although previous research has highlighted greater mortality in men in relation to cardiovascular disease, the effects in females should not be understated [21]. Investigating the influence of gender with N95 use and the subsequent development of cardiovascular disease is an area where further research would be needed.

Some studies, however, were identified, which showed contrasting findings on the effect of the N95 mask on heart rate with slight decreases in heart rate noted with the use of the N95 mask [13,14]. One of these studies reported a decrease in heart rate during an exercise stress test when comparing those who wore the N95 mask in comparison to those who did not [13]. The small sample size of only 20 participants in this study limits our ability to judge whether there is enough evidence that the N95 mask can reduce heart rate. In addition to this, Kampert et al. only looked at the effects during exercise and specifically during an exercise stress test [13]. Longer follow-up studies may help us get a more reflective picture of the effects of N95 masks on subjects during their daily course of activities. Yet, what appears to be clear, is that N95 masks appear to be safe and even under strenuous activity, they do not cause cardiovascular dysfunction [10].

Raised blood pressure is a major contributing factor in cardiovascular disease and if prolonged exposure exists to such elevated levels, serious detriment can occur to the body with consequences, including atrial fibrillation, coronary heart disease and heart failure amongst others [22]. Hypertension has been found to cause damage to the endothelium of vessels as well as commonly causing left ventricular hypertrophy, which results in an increase in demand for oxygen and can potentially cause myocardial ischaemia [23]. This clearly demonstrates the significance of blood pressure on cardiovascular health and thus makes it important to understand the effect N95 masks have on this parameter.

Research papers have shown that N95 respirators do not tend to influence blood pressure. This was well observed in one of the papers by Epstein D et al., where no significant changes in blood pressure were found during exercise whilst using an N95 mask [10]. The average systolic blood pressure without a mask was 143 ± 14 mmHg, whereas, with an N95, it was found to be 147 ± 16 mmHg, indicating the lack of any meaningful change in blood pressure. Though there is such little change in systolic blood pressure, one of the drawbacks of this study is that all the participants were healthy and relatively young, with a mean age of 34 ± 4 years [10]. Therefore, more investigations would be required to monitor the effect in those with conditions such as pre-existing hypertension and for those in an older age category where blood pressure naturally tends to be higher [24].

Although the full effect on blood pressure is unclear, there is evidence that N95 masks can reduce aortic augmentation pressure [18]. A reduced aortic augmentation pressure essentially means decreased stiffening of the aorta and has been associated with reduced cardiovascular events [25]. Although this is an important finding since it illustrates that there is potentially some benefit in N95 respirators reducing cardiovascular pathologies, more research is necessary to establish the relationship.

Individuals who experience symptoms such as dyspnoea are more likely to struggle with performing exercise and will have a significantly reduced exercise tolerance. The dyspnoeic effects on individuals could potentially promote a more sedentary lifestyle, in turn increasing the cardiovascular risk of such a population. Weight gain and a decreased level of fitness can arise as a subsequent consequence of such lifestyles, thus highlighting the importance of understanding the relationship between dyspnoea and the use of N95 respirators [26].

A key theme evident among the studies in the review is the effect N95 masks have on breathing. Multiple studies have shown that using an N95 respirator leads to greater rates of dyspnoea. One of these studies compared exercise tests performed prior to the pandemic without any mask against those performed during the pandemic whilst using an N95 mask [12]. A key finding from this cross-sectional study was that there was an increase in the number of exercise tests that were suspended due to dyspnoea compared to those tests conducted during the pandemic with an N95 mask [12].

This is a very relevant finding since it illustrates that N95 respirators can potentially lead to greater levels of shortness of breath. Although some of the participants in this study did have some underlying cardiovascular risk factors, including hypertension and diabetes, the majority of studies did not include participants with such conditions, yet noticed the effects of the N95 respirator on their breathing [8]. Consequently, those with existing pathologies, such as heart failure, would likely experience worsening of their symptoms whilst wearing an N95 mask, however, further trials would need to be conducted to find the exact effect.

End-tidal carbon dioxide has been found to be significantly elevated during the usage of N95 respirators whilst exercising [10]. Raised end-tidal carbon dioxide has been associated with an increase in cardiac output, which, in a chronic setting, has the potential to develop into high output heart failure [27,28]. Shechtman et al. also noted an increase in end-tidal carbon dioxide in healthcare workers who wore N95 masks for four hours [15]. Although there was some change in gas exchange parameters, it is worth noting that this was recorded via non-invasive capnography, and there is uncertainty whether masks affect this reading, thus the interpretation of this parameter must be done with care.

Rebmann et al. showed that after nurses had worn an N95 mask for a 12-hour shift, all physiological parameters were in the normal range, except a rise in carbon dioxide compared to baseline [17]. However, this was deemed not to be enough to cause clinical issues. As expected, there were some subjective symptoms, such as discomfort, lightheadedness, and dyspnoea but nothing to change the physiological parameters from baseline.

Several studies assessed whether the long-term use of N95 masks is safe and whether they can cause physiological changes that are clinically relevant. The overall consensus is that there are no attributable negative consequences from the use of N95 masks in the short to medium term. In one study by Roberge et al., healthy pregnant women (n = 22) and non-pregnant women (n = 22) had physiological measurements taken with and without wearing an N95 mask during exercise and postural activities over one hour [16]. There was no difference between both groups in heart rate, respiratory rate, oxygen saturation, transcutaneous carbon dioxide level, chest wall temperature, aural temperature, and subjective perceptions of exertion and thermal comfort. No significant effect on foetal heart rate was noted [16].

Low exercise tolerance has long been linked to conditions such as hypertension, diabetes and other cardiovascular events [29]. There is also concern that face masks can exacerbate or cause impairment in lung function (hypoxia and hypercapnia) during exercise and thereby decreasing exercise tolerance. A study by Ade et al. tested this with a surgical mask, flannel or N95 mask compared to no mask (control) [8]. None of these masks reduced sub-maximal or maximal exercise arterial O2 saturation (P = 0.744), but ratings of dyspnoea were significantly increased (P = 0.007) [8]. Exercise tolerance, however, remained unchanged as did key cardiovascular parameters, such as mean arterial pressure, stroke volume and cardiac output, during sub-maximal exercise. The limitation of the study is that it was a small sample size, consisting of young healthy recreational male and female exercisers [8]. As such, the results may be different for athletes, smokers or an older population with co-morbidities especially cardiovascular and respiratory pathology. The study also did not analyse the effect of mask-wearing with endurance-related exercises.

Traffic-related air pollution is well known to cause oxidative stress and vascular dysfunction and has pro-thrombotic effects [30]. Indeed, traffic exposure has been known to be a trigger for an acute myocardial infarction [30]. In a randomised controlled trial, near-roadway exposure to black carbon and overall particle count from exhausts was determined with and without an N95 mask [18]. In those without an N95 mask, there was arterial stiffening and a rise in aortic blood pressure, whereas in cases with an N95, there was a slight improvement in aortic parameters. However, as far as the black carbon and particle count (BC and PC) were concerned, there was no statistically significant change with or without an N95 mask and the P-value was 0.99 and 0.86, respectively [18]. This study helps reinforce the protective elements that the N95 mask can provide against exposure to air pollutants that have the potential to trigger cardiovascular disease.

Limitations

There are some limitations with this review that need to be highlighted. Usage of the N95 respirator has only become more common recently over the last two years following the COVID-19 pandemic. Consequently, it is a relatively novel area and there has not been extensive research on the topic making it difficult to be certain of their effects. Another key limitation is that the studies that have been conducted seem to focus heavily on young adults. The effects of N95 masks may differ significantly in children and in those of an older age who are more prone to suffering from cardiovascular pathologies. The studies also tend to focus on individuals wearing N95 respirators for a short period of time, mainly during exercise. However, the effects of wearing N95 masks consistently for a prolonged period of time may illicit a different physiological response. The overwhelming majority of studies appear to involve participants who do not have any underlying cardiovascular or respiratory disease and thus the effects of N95 respirators in these individuals would be understated. Again, barely any of the studies involved those with pre-existing risk factors, such as smoking and obesity, which are known to be associated with poorer cardiovascular outcomes. Finally, when analysing previous literature on the topic, one common problem that was observed was the confusion between N95 and FFP2 respirators. Some of the previous literature has used these synonymously despite there being some differences between the two. The lack of clarity around this is important to note since this could have influenced results in previous studies conducted.

## Conclusions

The findings of this review show that the usage of N95 masks has a multitude of effects on various parameters, which can influence the development of cardiovascular disease. Given the paucity of research within the area, further research with longitudinal and prospective cohort studies would help in providing evidence on whether N95 masks can help in the global battle against cardiovascular disease.

## References

[1] Howard J, Huang A, Li Z (2021). An evidence review of face masks against COVID-19. Proc Natl Acad Sci U S A.

[2] Li Y, Liang M, Gao L (2021). Face masks to prevent transmission of COVID-19: a systematic review and meta-analysis. Am J Infect Control.

[3] Kim MS, Seong D, Li H (2022). Comparative effectiveness of N95, surgical or medical, and non-medical facemasks in protection against respiratory virus infection: a systematic review and network meta-analysis. Rev Med Virol.

[4] Desai AN, Mehrotra P (2020). Medical masks. JAMA.

[5] Al-Kindi SG, Brook RD, Biswal S, Rajagopalan S (2020). Environmental determinants of cardiovascular disease: lessons learned from air pollution. Nat Rev Cardiol.

[6] Vohra K, Marais EA, Bloss WJ (2022). Rapid rise in premature mortality due to anthropogenic air pollution in fast-growing tropical cities from 2005 to 2018. Sci Adv.

[7] Liberati A, Altman DG, Tetzlaff J (2009). The PRISMA statement for reporting systematic reviews and meta-analyses of studies that evaluate health care interventions: explanation and elaboration. J Clin Epidemiol.

[8] Ade CJ, Turpin VG, Parr SK (2021). Does wearing a facemask decrease arterial blood oxygenation and impair exercise tolerance?. Respir Physiol Neurobiol.

[9] Ahmadian M, Ghasemi M, Nasrollahi Borujeni N (2022). Does wearing a mask while exercising amid COVID-19 pandemic affect hemodynamic and hematologic function among healthy individuals? Implications of mask modality, sex, and exercise intensity. Phys Sportsmed.

[10] Epstein D, Korytny A, Isenberg Y (2021). Return to training in the COVID-19 era: the physiological effects of face masks during exercise. Scand J Med Sci Sports.

[11] Han B, Zhao R, Zhang N (2021). Acute cardiovascular effects of traffic-related air pollution (TRAP) exposure in healthy adults: a randomized, blinded, crossover intervention study. Environ Pollut.

[12] Ilarraza-Lomelí H, Rojano-Castillo J, Carazo G, Flores S, Barrera-Ramírez C, Rius-Suárez MD, Ortega-Aranda H (2021). Cardiovascular evaluation using exercise testing performed with face masks during the COVID-19 pandemic. Arch Cardiol Mex.

[13] Kampert M, Singh T, Sahoo D, Han X, Van Iterson EH (2021). Effects of wearing an N95 respirator or cloth mask among adults at peak exercise: a randomized crossover trial. JAMA Netw Open.

[14] Su CY, Peng CY, Liu HL, Yeh IJ, Lee CW (2021). Comparison of effects of N95 respirators and surgical masks to physiological and psychological health among healthcare workers: a randomized controlled trial. Int J Environ Res Public Health.

[15] Shechtman L, Ben-Haim G, Ben-Zvi I (2022). Physiological effects of wearing N95 respirator on medical staff during prolong work hours in COVID-19 departments. J Occup Environ Med.

[16] Roberge RJ, Kim JH, Powell JB (2014). N95 respirator use during advanced pregnancy. Am J Infect Control.

[17] Rebmann T, Carrico R, Wang J (2013). Physiologic and other effects and compliance with long-term respirator use among medical intensive care unit nurses. Am J Infect Control.

[18] Morishita M, Wang L, Speth K (2019). Acute blood pressure and cardiovascular effects of near-roadway exposures with and without N95 respirators. Am J Hypertens.

[19] Perret-Guillaume C, Joly L, Benetos A (2009). Heart rate as a risk factor for cardiovascular disease. Prog Cardiovasc Dis.

[20] Hjalmarson Å (2007). Heart rate: an independent risk factor in cardiovascular disease. Eur Heart J Suppl.

[21] Bots SH, Peters SA, Woodward M (2017). Sex differences in coronary heart disease and stroke mortality: a global assessment of the effect of ageing between 1980 and 2010. BMJ Glob Health.

[22] Fuchs FD, Whelton PK (2020). High blood pressure and cardiovascular disease. Hypertension.

[23] Escobar E (2002). Hypertension and coronary heart disease. J Hum Hypertens.

[24] Anderson GH (1999). Effect of age on hypertension: analysis of over 4,800 referred hypertensive patients. Saudi J Kidney Dis Transpl.

[25] Chirinos JA, Zambrano JP, Chakko S (2005). Aortic pressure augmentation predicts adverse cardiovascular events in patients with established coronary artery disease. Hypertension.

[26] Katsoulis M, Stavola BD, Diaz-Ordaz K (2021). Weight change and the onset of cardiovascular diseases: emulating trials using electronic health records. Epidemiology.

[27] Weil MH, Bisera J, Trevino RP, Rackow EC (1985). Cardiac output and end-tidal carbon dioxide. Crit Care Med.

[28] Mehta PA, Dubrey SW (2009). High output heart failure. QJM.

[29] Georgiopoulou VV, Kalogeropoulos AP, Chowdhury R (2017). Exercise capacity, heart failure risk, and mortality in older adults: the Health ABC study. Am J Prev Med.

[30] Lucking AJ, Lundbäck M, Barath SL (2011). Particle traps prevent adverse vascular and prothrombotic effects of diesel engine exhaust inhalation in men. Circulation.

